# Barcoding Life to Conserve Biological Diversity: Beyond the Taxonomic Imperative

**DOI:** 10.1371/journal.pbio.1000417

**Published:** 2010-07-13

**Authors:** Ronnie Vernooy, Ejnavarzala Haribabu, Manuel Ruiz Muller, Joseph Henry Vogel, Paul D. N. Hebert, David E. Schindel, Junko Shimura, Gregory A. C. Singer

**Affiliations:** 1 Agriculture and Environment Program Area, International Development Research Centre (IDRC), Ottawa, Ontario, Canada; 2 Department of Sociology, University of Hyderabad, Hyderabad, India; 3 International Affairs and Biodiversity Program, Peruvian Society for Environmental Law (SPDA), Lima, Peru; 4 Department of Economics, University of Puerto Rico-Río Piedras, Puerto Rico, United States of America; 5 Biodiversity Institute of Ontario (BIO), University of Guelph, Guelph, Ontario, Canada; 6 Smithsonian Institution, National Museum of Natural History, Washington D.C., United States of America; 7 Secretariat of the Convention on Biological Diversity, Montreal, Quebec, Canada; 8 Biodiversity Institute of Ontario (BIO), University of Guelph, Ontario, Canada

## Abstract

Barcoding scientists aspire to adhere to the objectives of the Convention on Biological Diversity by promoting conservation, sustainability, and the equitable sharing of benefits arising from use of genetic resources. (Image: Juan Manuel Escalante, wwww.realitat.com)

In the 250 years since the Swedish scientist Carl Linnaeus first started classifying organisms, taxonomists have formally described roughly 1.7 million species. Although seemingly large, this number represents only a small fraction of the estimated tens of millions of species on the planet. Moreover, human activities are causing the extinction of species hundreds of times faster than the natural rate of extinction found in the fossil record. Fully one-third of all species on the planet may be gone by the end of this century—many without ever having been studied or, more importantly, protected [1].

DNA barcoding, developed in 2003 to identify species, has helped to rejuvenate taxonomic research. The science of taxonomy is key to understanding and monitoring biodiversity [2]. The technique is based on a simple but powerful observation: that sequence diversity, in short, standardized gene regions (i.e., DNA barcodes), can serve as a tool to identify known species and potentially discover new ones. Moreover, DNA barcoding allows researchers to develop a system for species identification based on digital characters, eventually allowing for automated identifications, thereby promising to improve the capacity to identify, monitor, and manage biodiversity, with profound societal and economic benefits. It also raises the possibility of identifying the vectors of zoonotic diseases as well as the disease organisms themselves.

Using DNA barcoding technology, researchers seek to build a library of short, standardized pieces of DNA from all of Earth's species—an admittedly massive undertaking that would enable the scientific community to quickly and accurately assess the Earth's biodiversity and monitor it over time [3]. The promise of this technology has captured the attention of the scientific community, government agencies, and the general public. Widespread support has led to nearly US$100 million in grants that have been used to mobilize a large research program in DNA barcoding and establish the Consortium for the Barcode of Life (CBOL; http://www.barcoding.si.edu), with 200 member organizations in 50 countries. A national research network in Canada has directed its efforts towards simplifying the protocols for DNA barcode acquisition, gathering barcode records, and developing the informatics platform needed for the curation and analysis of barcode records. The latter effort has led to the creation of BOLD, the Barcode of Life Data Systems (http://www.boldsystems.org), which now has more than 5,000 registered users and holds barcode records for more than 850,000 specimens, representing approximately 100,000 species. Individual organisms are placed in museum collections, and their extracted DNA resides in a secure repository, so that future generations can study them.

To coordinate these global efforts, an alliance of researchers and biodiversity conservation organizations plan to launch the International Barcode of Life Project (iBOL; http://www.ibolproject.org) in October 2010 the International Year of Biodiversity. The project will bring together 26 countries to broaden and strengthen DNA barcoding research with potential social, cultural, and economic, implications—direct and indirect—with a special focus on developing countries. Because the true stewards of biological diversity are at the local level, it is imperative that they be included in the process. This means obtaining consent from the competent authority before collecting specimens and barcoding, sharing the resulting data with local people, and building capacity to use the new knowledge generated. Barcoding appears to fall under the scope of “access to genetic resources and the fair and equitable sharing of benefits” in the Convention on Biological Diversity. Barcoders must disclose up-front foreseeable consequences of their research (both intended and unintended) so that governments at national and state levels can develop informed policies.

To date, researchers in developed countries have largely performed DNA barcoding, even though most of the Earth's biodiversity is found in the tropical and subtropical regions. The iBOL aims to rectify this situation by collaborating with researchers and local communities in developing countries to retrieve DNA barcodes from 5 million specimens representing 500,000 of Earth's species within the first five years of its operations. Work will focus on taxonomic groups targeted for analysis by iBOL because they deliver key ecosystem services (e.g., pollinators), because they are pests (e.g., termites), because they are harvested (e.g., fishes, forest trees), or because they are important targets for conservation programs (e.g., mammals, reptiles). A number of teams in developing countries will deploy scientific expertise and the built-up barcode library to apply barcoding as a tool for environmental or ecosystem management.

## Building Partnerships

Because many developing countries lack the resources and scientific capacity to perform DNA sequencing, iBOL has developed a tiered participation structure to enhance inclusiveness. It consists of national, regional, and central nodes. iBOL will bolster scientific capacity by providing the funding necessary to train young scientists. In addition, it will broaden exposure to barcode technology by delivering training sessions and by launching projects that validate the practical implications of barcode technology. The database of Earth's biodiversity arising from iBOL will be free for the world to access and use. Inasmuch as commercial applications may arise from barcoding technologies and their use, iBOL is committed to ensuring that citizens of all nations have an equal chance to take advantage of any opportunity [4]. iBOL adheres to the three main objectives of the Convention on Biological Diversity (CBD): conservation of biodiversity; sustainable use of biodiversity; and the fair and equitable sharing of the benefits arising from the use of genetic resources.

There are many benefits to iBOL participation for capacity building at a national level in countries that harbor much of the biodiversity that barcoders are interested in, such as the training of students in state-of-the-art field collection and laboratory techniques and the development of basic science. Nevertheless, history is filled with countless examples of other scientific projects where—even with the best of intentions—benefits derived from biological materials were never shared with the countries of origin. Governments of developing countries are understandably skeptical of scientific endeavors originating from the developed countries, sometimes resulting in strict laws—as in India—where foreign access to biological materials is all but impossible.

Perhaps because iBOL and barcoding are more driven by natural scientists, most barcoders have thus far taken a rather naive stance on issues of access and benefit sharing (ABS) that belong more to the spheres of policy and law. Where difficulties have been encountered in obtaining specimens from certain countries with strict biodiversity export rules (e.g., Brazil and India), barcode projects have been delayed or limited to small-scale efforts. However, iBOL recognizes that such a strategy is not sustainable as the endeavor continues to grow. For this reason, iBOL together with CBOL asked experts at a recent conference (http://www.dnabarcodes2009.org/) to draft position papers about access and benefit sharing as they relate to the iBOL project. Summaries of three key perspectives, relating barcoding to the Convention on Biological Diversity, economics, and the sociological implications in one region are provided in Boxes 1–3, respectively. They serve to illustrate the complexity and contention surrounding some of the major issues involved. The three presentations from the Third International Barcode of Life Conference in Mexico City, November 2009, are freely available as video through the iBOL site at http://vimeo.com/user2807308.

**Box 1. *The Convention on Biological Diversity: Access and Benefit Sharing Principles in the Context of Barcoded Genetic Information*, by Manuel Ruiz Muller (Director of the International Affairs and Biodiversity program of the Peruvian Society for Environmental Law, Lima, Peru) [6]**

Since 1993, the year when the Convention on Biological Diversity (CBD) entered into force, the “common heritage of mankind” principle has given way to the recognition of sovereignty over genetic resources. Laws and regulations now affirm state rights over genetic resources and seek to establish legal regimes based on access to genetic resources and the fair and equitable sharing of benefits (ABS). These legal frameworks cover mostly animal, plant, and microbial genetic resources as defined by the CBD.

Parallel advances in modern biotechnology (e.g., cloning) and whole new disciplines—genetic engineering, genomics, proteomics—have radically transformed research and development processes. Yet, these technologies and new scientific disciplines are largely unaccounted for in policy and legal debates concerning ABS. Barcoding is exemplary of this phenomenon.

The CBD defines genetic resources very broadly as “genetic material of actual or potential value” and genetic material as “any material of plant, animal, microbial, or other origin containing functional units of heredity.” This “potential value” is rapidly being realized in many fields, including pharmaceuticals, medicine, and plant-breeding.

Gene libraries and rich, sophisticated databases are transforming the way scientists undertake routine research. For example, anyone with the requisite scientific skills, a good laptop computer and a reliable Internet connection, can easily access, screen, and transform genetic information into potentially useful innovations. Indeed, new theories, tools, and technologies are changing the way biological classification takes place. As soon as DNA or genes are involved, that is “the functional units of heredity,” the CBD ABS policy and legal principles become relevant.

Seeking a balance between private rights and sovereignty is one of the driving forces behind CBD policy and legal developments, as evidenced in access legislation and defensive protection measures, including the protection of traditional knowledge. In each case, the nature and timing of benefits will depend on the policies and regulations set by states, and agreements reached between states, researchers, companies, and indigenous people.

To date, the seemingly endless debate about ABS in the CBD eclipses an often overlooked set of provisions of the CBD that refers to conservation per se and the need to understand biodiversity dynamics and species at the ecosystem level. Taxonomy, in this regard, is a critical discipline that serves conservation and may also play an important role in profit, commercial, or industrial endeavours.

Encouragingly, one notes that members of iBOL “…are committed to the regulatory framework established under the CBD” and expressly indicate that “transactions between iBOL members will respect all restrictions with respect to biomaterials transfers.” However, it is far from clear what exactly this commitment means and how it will be put into practice. A global project of this magnitude should reflect upon the potential social, cultural, and economic, implications—direct and indirect—of its activities, particularly on developing countries. The main concern is whether and how CBD principles are pertinent or even relevant to work and activities undertaken by iBOL. Here is a preliminary list of questions for the stakeholders in iBOL:

- Do ABS principles apply to iBOL activities?
- How will developing countries participate in iBOL?
- Do iBOL activities affect national sovereignty of countries of origin?
- Is the sovereignty of countries of origin being affected when genetic resources are accessed from Barcode of Life databases?
- How might the cultural rights of indigenous people be affected by iBOL?
- What benefits can be shared with countries of origin?
- How with those benefits be shared?
- And, lastly, are commercially or industrially oriented uses of iBOL services and products envisioned? If so, are there limitations, guidelines or other orienting principles?

Because a diversity of opinions will emerge in response to the above questions, negotiation appears to be paramount.

**Box 2. *iBOL as an Enabler of ABS and ABS as an Enabler of iBOL*, by Joseph Henry Vogel (Professor of Economics, University of Puerto Rico-Río Piedras, USA) [6]**

“Thinking like an economist” is the mantra of my profession and I cringe whenever I hear it. I count myself among the dissidents who believe that “thinking like an economist” has enabled the destruction of biological communities, both human and non-human [7]. Nevertheless, I would be the first to say “let's not throw the baby out with the bathwater.” Much of the discussion about the iBOL proceeds as if formal economics did not exist. Since the ratification of the United Nations Convention on Biological Diversity (CBD) in 1993, I have grown inured to the lack of *any* economic thinking when the Conference of the Parties (COP) meets to discuss access and benefit sharing (ABS) [8]. Insisting on thinking like an economist, I hope to show that a baby can still emerge from the confluence of ABS and iBOL.

Ever since Adam Smith, economics has been associated with selfish interests. How do we align the interests of industry which researches and develops genetic resources with those of countries which decide the fate of those resources? [9] The answer from the CBD is S-O-V-E-R-E-I-G-N-T-Y. Various articles overturn “the common heritage of mankind” and allow countries to negotiate bilaterally. Who could object? I did and let me explain by way of an analogy [10].

In the streets of the developing world, a thriving market exists in pirated movies. The hawker typically asks $1 per DVD which is 5% of the retail price. Why not $19, $18…or even $2? The answer is competition. Each hawker underbids other hawkers and the price falls to the marginal costs of hawking. No monopoly rent is ever paid to the creator of the artificial information, namely, Hollywood.

The same holds true for natural information. When reported, royalties are typically 1% or less. Most metabolites are diffused across species and species, across political boundaries. Each country underbids other countries and ABS falls to the marginal costs of consummating a Material Transfer Agreement (MTA). No rent is ever paid to the conservationist of natural information—the countries of origin. Thinking like an economist, the only way to capture a rent (e.g., 15%) is through the cartelization of genetic resources [11].

How much would each country get? A simple solution is a share proportional to the habitat. If Brazil occupies 56% of the Amazon and Ecuador, 2%, the former would get 56% of the royalty and the latter, 2% for a metabolite distributed throughout the basin. Cartelization with a concomitant disclosure requirement of species in patent applications would obviate MTAs and allow the free flow of genetic resources.

iBOL can become the enabler for ABS but does ABS enable iBOL? The answer is money. Many metabolites are so widely distributed that the cumulative costs of determining each country's fair share would outstrip the sum collected. In such cases, the royalties “should be used to diminish the fixed costs of the gargantuan database” [12]. I penned those words in 1992 long before I imagined that iBOL would emerge as a gargantuan database.

Will iBOL support a biodiversity cartel in the ongoing COP discussions about an “International Regime on ABS?” I am hopeful, not because I believe that iBOL will do the right thing—people seldom do. My reason for hope is that doing the right thing behooves iBOL, materially so. Adam Smith's most famous phrase about the butcher, the brewer and the baker, is still apt. It will not be from the benevolence of iBOL that iBOL enables fair and equitable ABS, but from regard to its own interest. Now, what could be more economic in thinking than that?

**Box 3. *DNA Barcoding: Society and technology dynamics in the Indian context*, by Ejnavarzala Haribabu (Professor of Sociology, University of Hyderabad, India) [6]**

India is a “mega hot spot” of biodiversity. A culture of conservation has emerged over the years as part of a utilitarian and aesthetic value system where households and communities conserve the germplasm of crop and medicinal plants as well as horticulture and some species of animals in situ. In the absence of written rules, the conservation and use of biodiversity have been traditionally regulated by religious norms and sanctions. Over time, communities acquired empirical knowledge about the utility of various plant species and animals and developed local taxonomies that they share.

The Plant Varieties and Farmers' Rights Act in 2001 and the National Biological Diversity Act in 2002 gave effect to the CBD in the Indian context and now govern ABS issues. The National Biological Diversity Act (NBDA) does not allow foreign nationals and non-resident Indians to carry out biodiversity research or develop and access genetic resources without prior permission of the National Biodiversity Authority (NBA). No person can apply for any intellectual property right in or outside India for any invention based on any research or information on a biological resource obtained from India without obtaining the prior approval of the NBA. The Authority may impose a benefit sharing fee or a royalty, or both. However, the recent amendment of the Act notified through the Gazette of India by the Ministry of Environment and Forests on November 8, 2006, allows access to biodiversity for collaborative projects in the area of taxonomic studies subject to the approval of concerned Departments/Ministries of the Government of India.

Any attempt to barcode species for research purposes—whether commercial or not—requires the prior informed consent of the custodians or stewards. The normative basis of terms of consent differs in the two situations. As of this writing, the Act seems to emphasise access and benefit sharing in the context of commercial research. Nevertheless, a series of questions regarding non-commercial research typical of iBOL arises: what are the norms that should govern access that (a) promotes non-commercial research, (b) protects the national sovereignty over genetic resources, and (c) ensures non-monetary benefits, if any? Schindel et al. [4] point out the tangible indicators of distinguishing non-commercial research from commercial research [13]. These include the generation of new knowledge, the collection of reference specimens in the public domain, and capacity building, particularly in developing countries. Regarding the latter, one can say that in India there are qualified and competent scientists and well endowed molecular biology research institutions.

Because the Act regulates access to commercial research, there is a need to examine whether or not the existing provisions and the recent amendments made to the provisions of the Act, are adequate to ensure that the genetic resources accessed for non-commercial research are not used for commercial research at a later date, and are not shared with a third party. Further, norms for material transfer agreements must protect national interests; and communities should participate in negotiations regarding access and benefit-sharing.

As a part of this process of democratization of barcoding technology, efforts should be made to seek the consent of local communities and build their capacities to use barcoded information for monitoring and developing biodiversity. Communities should be informed about the benefits of barcoding and take part in decisions regarding what species are to be barcoded.

## The Challenges Ahead

Article 2 of the Convention on Biological Diversity defines genetic resources “as genetic material of actual or potential value,” where genetic material means “any plant, animal, microbial, or other origin containing functional units of heredity.” Yet, the genetic material in food, fibers, and extracts—containing functional units of heredity—flow freely across borders while well-meaning scientific endeavors are blocked.

The interpretation of the definition of genetic resources is still an area of active debate within the negotiations of the International Regime on access and benefit sharing under the CBD, which is to be adopted at the 10th meeting of the Conference of the Parties, in October 2010, in Japan [5]. iBOL must attempt to influence that debate to ensure a set of recommendations that protects biodiversity-rich nations while simultaneously avoiding the inhibition of scientific progress.

Recognizing that the road ahead will not be smooth as access and benefit sharing issues remain highly contentious, there are steps that iBOL can take to ensure that roadblocks are minimized:

- First and foremost, DNA barcoding needs to be recognized as non-commercial research, like most of taxonomy. It offers significant non-monetary benefits that will be shared openly with countries that provide access to their biodiversity by participating in iBOL.
- Second, it is necessary to formalize what it means for a nation to participate in iBOL. ABS concerns must be clearly laid out and guidelines for the commercial or industrial use of iBOL-related products and services should be established.
- To the extent possible, iBOL must determine from the outset foreseeable consequences of the project (both intended and unintended) so that governments can develop informed policies.
- Finally, although it is critical to engage national and state governments, the true stewards of biological diversity lie at the local level and must be included in the process. This not only means obtaining consent from the competent authority before collection and DNA barcoding, but also sharing the resulting data with local communities and training them in its use.

## Abbreviations

ABS: access and benefit sharing (of genetic resources)
BOLD: Barcode of Life Data Systems
CBD: Convention on Biological Diversity
CBOL: Consortium for the Barcode of Life
COP: Conference of the Parties
DNA: deoxyribonucleic acid
iBOL: International Barcode of Life (project)
MTA: Material Transfer Agreement
NBA: National Biodiversity Authority (India)
NBDA: National Biodiversity Act (India)
